# Adaptive class rebalancing and spatial-aware metadata fusion for long-tailed skin lesion classification

**DOI:** 10.3389/fmed.2026.1841617

**Published:** 2026-06-23

**Authors:** Tianming Ma, Xianwei Han, Guijun Liu

**Affiliations:** 1 The Second Affiliated Hospital of Heilongjiang University of Chinese Medicine, Harbin, China; 2 Department of Dermatology, Shenyang Seventh People's Hospital, Shenyang, China; 3 Heilongjiang University of Chinese Medicine, Harbin, China

**Keywords:** class imbalance, contrastive learning, cross-modal fusion, focal loss, skin lesion classification, vision transformer

## Abstract

**Introduction:**

Dermoscopic skin lesion classification is challenged by class imbalance and the underutilization of clinical metadata, limiting the diagnostic reliability of existing deep learning systems.

**Methods:**

Building upon a Vision Transformer baseline that fuses dermoscopic images with patient metadata via cross-modal attention, we introduce three complementary improvements: class-balanced focal loss with square-root effective-number sampling, a patch-level cross-attention metadata-guided attention module, and supervised contrastive regularization. The extended model is evaluated on the ISIC 2019 and BCN 20000 datasets.

**Results:**

Ablation studies demonstrate consistent improvements. On the ISIC 2019 validation set, the full model achieves a Macro AUC of 0.9613 and a Macro balanced accuracy of 0.8426, with strong performance on rare categories such as dermatofibroma (AUC = 0.991) and vascular lesions (AUC = 0.999).

**Discussion:**

Principled loss design, spatially aware fusion, and representation-level regularization jointly improve the robustness of skin lesion classification under extreme class imbalance.

## 1 Introduction

Skin cancer is one of the most common malignancies worldwide, with incidence rates rising steadily, particularly in fair-skinned populations (1). Early detection of malignant melanoma and other high-risk lesions is crucial, as patient prognosis deteriorates dramatically once the disease progresses to advanced stages. Dermoscopy has therefore become a standard non-invasive imaging tool in clinical practice, providing magnified and polarized views that reveal sub-surface skin structures invisible to the naked eye (2). In parallel, deep learning has demonstrated dermatologist-level performance across several skin lesion classification benchmarks (3, 4), spurring intense interest in computer-aided decision support systems for dermatology.

Building on these advances, the International Skin Imaging Collaboration (ISIC) has organized a series of public challenges, releasing large-scale dermoscopic datasets to standardize lesion diagnosis (5, 6). The ISIC 2019 classification task is particularly significant from a clinical perspective: it covers eight diagnostic categories (ranging from common nevi to rare malignancies), aggregates data from multiple international centers, and emphasizes performance on rare but critical classes via balanced accuracy metrics (7). Furthermore, the challenge encourages the use of patient-level metadata—such as age, sex, and anatomical site—alongside dermoscopic images. While prior works have explored CNN ensembles (7, 8) and multimodal transformers (9, 10), most state–of–the–art systems either rely on heavy computational overhead or treat metadata as a simple auxiliary vector concatenated to image embeddings, failing to explicitly model the complex interactions between visual and clinical cues.

**Figure 1:**
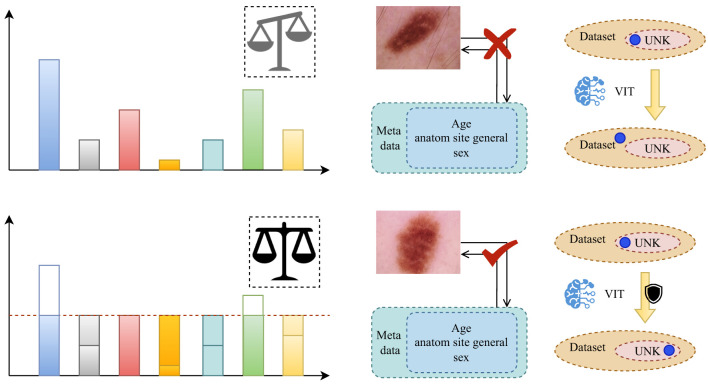
Motivation and core contributions of ISIC-ViT. The **top row** illustrates three key challenges in skin lesion classification: **(Left)** severe class imbalance leading to biased learning; **(Middle)** ineffective fusion between images and metadata (marked by the cross); and **(Right)** the risk of misclassifying “Unknown” (UNK) samples in open-set scenarios. The **bottom row** presents our corresponding solutions: Class-Aware Optimization (CAO) rebalances the training focus (indicated by the leveled bars); the Metadata-guided attention module (MAM) enables deep cross-modal interaction (marked by the checkmark); and the UNK-aware Decision Rule (UDR) acts as a safeguard (symbolized by the shield) to correctly reject unknown categories.

However, a closer look at the ISIC 2019 setting reveals several persistent challenges that remain insufficiently addressed by existing methods, as illustrated in the top row of Figure 1. First, the class distribution is highly long-tailed Figure 1 (left panel, top), with common benign nevi outnumbering rare malignant categories (e.g., vascular lesions) by orders of magnitude. This imbalance biases standard empirical risk minimization toward frequent classes, sacrificing sensitivity on rare diagnoses. Second, simple concatenation of metadata and image features Figure 1 (middle panel, top) often fails to capture meaningful correlations. Since age and site distributions differ markedly between classes, naive fusion can cause networks to either overfit spurious shortcuts or effectively ignore the metadata stream. Finally, the official test set introduces an open-set scenario with an “unknown” (UNK) category. Closed-set classifiers Figure 1 (right panel, top) are forced to assign these outliers to known categories, resulting in high-confidence errors (7). These factors make it non-trivial to design a unified architecture that is both clinically accurate and robust to real-world distribution shifts.

In this work, we address these hurdles by proposing ISIC-ViT, a multi-modal Vision Transformer framework tailored for robust skin lesion classification. As shown in the bottom row of Figure 1, our approach introduces three targeted components to resolve the aforementioned limitations. To counter biased learning, we design a Class-Aware Optimization (CAO) strategy Figure 1 (left panel, bottom) that combines balanced sampling with a label-smoothed, cost-sensitive loss to equalize the model's focus across all categories. To improve feature interaction, we replace naive concatenation with a Metadata-guided attention module (MAM) Figure 1 (middle panel, bottom). By projecting image and metadata into a shared latent space and performing cross-modal self-attention, MAM allows clinical attributes to adaptively re-weight visual features. To handle open-set risks, we implement an UNK-aware Decision Rule (UDR) Figure 1 (right panel, bottom), which employs a confidence threshold to safely reject out-of-distribution lesions during inference. Together with an Exponential Moving Average (EMA) stabilization, these modules form a coherent system that aligns strictly with the official ISIC 2019 protocols.

Building upon these foundations, we further extend ISIC-ViT with three complementary improvements. First, we replace the label-smoothed class-weighted cross-entropy with a class-balanced focal loss (11, 12) that jointly accounts for effective sample numbers and modulates the loss contribution of easy vs. hard examples, paired with a square-root effective-number sampling strategy for smoother gradient flow. Second, we upgrade the MAM to a patch-level cross-attention MAM that allows metadata embeddings to attend to all spatial patch tokens from the ViT backbone, capturing fine-grained spatial correlations between clinical context and local image regions. Third, we add a supervised contrastive regularization (13) term that encourages intra-class compactness and inter-class separation in the shared fusion space, further improving the discriminability of the learned representations.

Our main contributions are summarized as follows:

- We present ISIC-ViT, a unified multi-modal architecture that jointly models dermoscopic images and patient metadata via a lightweight cross-modal attention mechanism, explicitly capturing the interaction between visual appearance and clinical context.
- We develop a comprehensive training strategy to mitigate the severe class imbalance of ISIC 2019. By integrating class-balanced sampling, metadata-aware fusion, and EMA stabilization, our method achieves consistent gains over strong CNN and transformer baselines.
- We extend the original framework with class-balanced focal loss, patch-level cross-attention fusion, and supervised contrastive regularization, yielding further improvements in both ranking quality (Macro AUC) and balanced accuracy across the ISIC 2019 and BCN 20000 benchmarks.
- We conduct extensive experiments, including comparison with baselines, ablation studies on two datasets, and detailed per-class analysis, demonstrating the effectiveness and generalizability of the proposed approach.

## 2 Related work

We briefly review the literature from three aspects, i.e., deep learning for skin lesion classification, multi-modal/transformer-based dermatology models, and loss functions for long-tailed recognition.

### 2.1 Deep learning for skin lesion classification

Early studies on automated skin cancer screening mainly relied on handcrafted features and shallow classifiers, which were quickly surpassed by convolutional neural networks (CNNs). Esteva et al. (3) first demonstrated that an end-to-end CNN trained on large-scale clinical images could reach dermatologist-level performance for distinguishing malignant from benign skin lesions. Subsequent studies confirmed the effectiveness of deep CNNs on dermoscopic benchmarks such as the ISIC challenges (6), where residual networks, Inception-style architectures, and ensemble models became dominant solutions. Haenssle et al. (14) systematically compared dermatologists and a CNN for melanoma recognition and showed that machine performance was competitive even in realistic reading settings, further motivating research into reliable computer-aided diagnosis tools. More recent articles focus on stronger backbones and training strategies: Gessert et al. (7) use ensembles of multi-resolution EfficientNets with extensive data augmentation to achieve strong ISIC 2019 performance, while other studies investigate class-imbalance handling, calibrated probabilities, and robust uncertainty estimation. CNN-based feature extractors are also widely used in related recognition problems such as vehicle re-identification (15), where residual and transformer-style backbones are combined with task-specific modules. Despite these advances, many lesion-classification systems still operate on image pixels alone, or treat non-image information in an *ad-hoc* way, and they rarely consider open-set evaluation protocols with “unknown” classes as in our ISIC 2019 setting.

### 2.2 Multi-modal and transformer-based dermatology models

In clinical practice, dermatologists routinely combine visual inspection of dermoscopic or clinical photographs with structured patient information such as age, sex, and lesion location. A growing body of work, therefore, explores multi-modal models that fuse images with metadata. Nunnari et al. (16) study different strategies for combining pixels and patient metadata in CNN-based skin-lesion classifiers, including early fusion, late fusion, and intermediate concatenation. Gessert et al. (7) augment EfficientNet ensembles with meta-features (age, sex, and lesion site), showing that even simple concatenation can yield noticeable gains on ISIC 2019. In parallel, vision transformers (ViTs) have emerged as powerful alternatives to CNNs. Dosovitskiy et al. (17) show that pure transformer architectures can achieve competitive image classification accuracy when trained on large-scale data, inspiring applications in medical imaging. For skin lesion analysis, recent models such as SkinDistilViT (18) adapt ViT backbones (and their distilled variants) to dermoscopy images and report improvements over conventional CNNs, but they typically operate on images only and use standard softmax training without explicit consideration of metadata, class imbalance, or open-set recognition. Compared with these works, our method uses a ViT-B/16 backbone to encode dermoscopic images while learning a compact embedding of patient metadata, and then performs cross-modal fusion with multi-head attention. This design allows the model to capture not only strong visual semantics but also fine-grained dependencies between lesion appearance and clinical context, and it is evaluated under the official ISIC 2019 scoring protocol that emphasizes balanced accuracy and unknown-class handling.

### 2.3 Loss functions for long-tailed recognition

Class imbalance is a persistent challenge in medical image classification, and a rich body of work has investigated loss-level solutions. Lin et al. (12) introduced focal loss, which down-weights the contribution of well-classified (easy) examples by modulating the standard cross-entropy with a factor ${\left(1-p_{t}\right)}^{\gamma }$, thereby focusing optimization on hard samples. Cui et al. (11) proposed class-balanced (CB) loss, which re-weights each class according to its *effective number of samples* $E_{c}=\left(1-\beta^{N_{c}}\right)/\left(1-\beta \right)$, providing a theoretically motivated alternative to simple inverse-frequency weighting. The combination of CB weighting with focal loss (CB Focal Loss) has proven effective in long-tailed visual recognition. In parallel, Khosla et al. (13) extended the self-supervised contrastive learning framework of SimCLR to the supervised setting, where positive pairs are drawn from the same class and negatives from different classes. Supervised contrastive (SupCon) loss has been shown to produce representations with better intra-class compactness and inter-class separation, which is particularly beneficial when class boundaries are subtle—as is often the case in dermoscopic images. In this work, we integrate CB focal loss for the primary classification objective and add a SupCon regularizer in the shared fusion space, jointly addressing both the label-distribution skew and the representation-quality bottleneck.

## 3 Method

**Figure 2:**
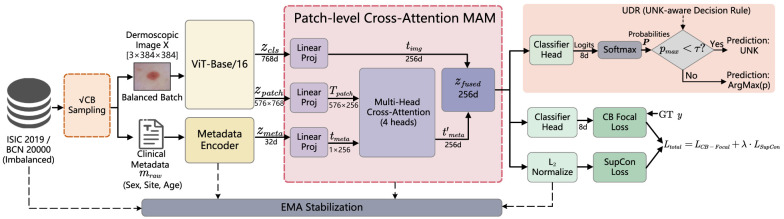
Pipeline of the proposed ISIC-ViT framework for ISIC 2019 skin lesion classification. The baseline branch adopts a ViT-Base/16 backbone to extract a global dermoscopic representation. A metadata-guided cross-modal attention module (MAM) performs interaction between image and clinical metadata. A CAO stabilizes training via balanced sampling and EMA. An UDR handles the unknown category at test time. All components are optimized jointly using a label-smoothed, class-weighted cross-entropy loss.

To address the ISIC 2019 skin lesion classification task, we design a Vision Transformer-based framework, termed ISIC-ViT, as illustrated in Figure 2. Given a dermoscopic image and its associated clinical metadata, ISIC-ViT first extracts a global image representation using a ViT-Base backbone, which serves as our baseline classifier. On top of this baseline, we introduce three lightweight yet effective components: a Metadata-guided cross-modal Attention Module (MAM), a CAO, and an UDR. We further extend the framework with a class-balanced focal loss with square-root effective-number sampling (Section 3.6), a patch-level cross-attention MAM (Section 3.7), and a supervised contrastive regularization term (Section 3.8). In the following, we describe the baseline and each component in detail.

### 3.1 Baseline: ViT-based ISIC classifier

Let **I** ∈ ℝ^*H* × *W* × 3^ denote a dermoscopic RGB image. After pre-processing and resizing, we obtain an input tensor, as shown in Equation 1:


$$\begin{array}{l} X\in {\mathbb{R}}^{3\times 384\times 384}, \end{array}$$ (1)

where 3 denotes the number of color channels, and 384 is the spatial resolution along both height and width. Each image is associated with a ground-truth diagnostic label, as shown in Equation 2:


$$\begin{array}{l} y\in \left\{0,1,\ldots ,C-1\right\}, \end{array}$$ (2)

where *C* = 8 corresponds to the known ISIC 2019 lesion categories: melanoma (MEL), nevus (NV), basal cell carcinoma (BCC), actinic keratosis (AK), benign keratosis (BKL), dermatofibroma (DF), vascular lesions (VASC), and squamous cell carcinoma (SCC).

We adopt the vit_base_patch16_384 backbone from timm as our baseline image encoder. The backbone is initialized from ImageNet-pretrained weights and configured with num_classes = 0 so that it only outputs latent features. Given an input tensor **X**, the Vision Transformer first partitions it into non-overlapping patches of size 16 × 16, projects each patch into a token embedding, and adds a learnable [CLS] token. After passing through multiple self-attention layers, we obtain a CLS embedding, as shown in Equation 3:


$$\begin{array}{l} z_{\text{img}}=f_{\text{ViT}}\left(X\right)\in {\mathbb{R}}^{D_{\text{img}}}, \end{array}$$ (3)

where *f*_ViT_(·) denotes the ViT backbone and *D*_img_ = 768 is the dimensionality of the CLS token.

In the baseline classifier, we map this global image representation **z**_img_ directly to class logits via a linear classifier, as shown in Equation 4:


$$\begin{array}{l} \ell^{\text{base}}=W_{\text{base}}z_{\text{img}}+b_{\text{base}}\in {\mathbb{R}}^{C}, \end{array}$$ (4)

where $W_{\text{base}}\in {\mathbb{R}}^{C\times D_{\text{img}}}$ and ${\text{b}}_{\text{base}}\in {\mathbb{R}}^{C}$ are learnable parameters.

The corresponding baseline prediction is obtained by softmax normalization, as shown in Equation 5:


$$\begin{array}{l} p_{c}^{\text{base}}=\frac{\exp\left(\ell_{c}^{\text{base}}\right)}{\sum_{j=0}^{C-1}\exp\left(\ell_{j}^{\text{base}}\right)},\;c\in \left\{0,\ldots ,C-1\right\}, \end{array}$$ (5)

where $p_{c}^{\text{base}}$ denotes the predicted probability that **I** belongs to class *c*. This baseline already benefits from strong global context modeling via the Transformer backbone, but it ignores clinical metadata and does not explicitly address class imbalance or the UNK category, motivating the following components.

### 3.2 Metadata-guided attention module (MAM)

Dermoscopic images are often accompanied by clinical metadata, such as patient sex, anatomical site, and approximate age. These attributes provide complementary cues that are highly correlated with lesion distribution and morphology. To exploit such information, we encode metadata into a low-dimensional embedding and design a lightweight cross-modal attention module to fuse it with the image representation.

#### 3.2.1 Metadata encoding

Let the raw metadata is defined in Equation 6:


$$\begin{array}{l} m_{\text{raw}}=\left(\text{sex},\;\text{anatom\_site},\;\text{age}\right), \end{array}$$ (6)

where sex takes values in {male, female, unknown}, anatom_site belongs to a set of 9 known anatomical locations plus an “unknown” category, and age is an approximate integer age with possible missing values.

We convert **m**_raw_ into a fixed-length numeric vector, as shown in Equation 7:


$$\begin{array}{l} m\in {\mathbb{R}}^{D_{\text{meta}}},\;D_{\text{meta}}=15, \end{array}$$ (7)

by concatenating: (i) a 3-dimensional one-hot encoding for sex, (ii) a 10-dimensional one-hot encoding for anatomical site (9 known categories and 1 “site_unknown” category), and (iii) a 2-dimensional age representation containing a normalized age value ã = age/100 and a binary missing indicator (1 if age is missing, 0 otherwise).

We then transform **m** using a two-layer MLP, as shown in Equation 8:


$$\left\{ \begin{array}{l} h_{1}\;=\text{BN}(\sigma (W_{1}m+b_{1})),\\ h_{2}\;=\text{Dropout}(h_{1};p=0.1),\\ z_{\text{meta}}\;=\text{BN}(\sigma (W_{2}h_{2}+b_{2})), \end{array}\right.$$ (8)

where $W_{1}\in {\mathbb{R}}^{64\times 15}$ and $W_{2}\in {\mathbb{R}}^{32\times 64}$ are learnable weight matrices, **b**_1_ and **b**_2_ are bias vectors, σ(·) denotes the ReLU activation, and BN is batch normalization.

The resulting metadata embedding ${\text{z}}_{\text{meta}}\in {\mathbb{R}}^{D_{\text{meta-emb}}}$ with *D*_meta-emb_ = 32 captures clinically relevant information in a compact form.

#### 3.2.2 Cross-modal attention fusion

To enable interaction between dermoscopic and clinical information, we project both image and metadata features into a common fusion space of dimension *D*_f_ = 256, as shown in Equation 9:


$$\{\begin{array}{ll} t_{\text{img}} & =W_{\text{img}}z_{\text{img}}+b_{\text{img}}\in {\mathbb{R}}^{D_{\text{f}}},\\ t_{\text{meta}} & =W_{\text{meta}}z_{\text{meta}}+b_{\text{meta}}\in {\mathbb{R}}^{D_{\text{f}}}, \end{array}$$ (9)

where $W_{\text{img}}\in {\mathbb{R}}^{D_{\text{f}}\times D_{\text{img}}}$, $W_{\text{meta}}\in {\mathbb{R}}^{D_{\text{f}}\times D_{\text{meta-emb}}}$, and ${\text{b}}_{\text{img}},{\text{b}}_{\text{meta}}\in {\mathbb{R}}^{D_{\text{f}}}$ are learnable parameters.

We then construct a sequence of two tokens, as shown in Equation 10:


$$\begin{array}{l} T=\left[\begin{array}{c} t_{\text{img}}^{⊤}\\ t_{\text{meta}}^{⊤} \end{array}\right]\in {\mathbb{R}}^{2\times D_{\text{f}}}, \end{array}$$ (10)

where the first row corresponds to the image token and the second row to the metadata token.

A 4-head multi-head self-attention layer is applied over this two-token sequence, as shown in Equation 11:


$$\begin{array}{l} T^{\prime }=\text{MHA}\left(T,T,T\right)\in {\mathbb{R}}^{2\times D_{\text{f}}}, \end{array}$$ (11)

where MHA(·) denotes the standard multi-head attention operation with queries, keys, and values all equal to **T**.

This operation allows the model to attend between the dermoscopic representation and metadata, learning how clinical attributes modulate visual features.

We aggregate the attended tokens via average pooling and apply layer normalization, as shown in Equation 12:


$$\begin{array}{l} z_{\text{fused}}=\text{LN}\left(\frac{1}{2}\overset{2}{\underset{i=1}{\sum }}T_{i}^{\prime }\right)\in {\mathbb{R}}^{D_{\text{f}}}, \end{array}$$ (12)

where ${\text{T}}_{i}^{\prime }$ denotes the *i*-th row of **T**′ and LN denotes layer normalization.

Finally, the fused representation is passed through a classification head, as shown in Equation 13:


$$\{\begin{array}{ll} h_{\text{cls}} & =\text{Dropout}(\text{BN}(\sigma (W_{3}z_{\text{fused}}+b_{3}))),\\ \ell & =W_{4}h_{\text{cls}}+b_{4}\in {\mathbb{R}}^{C}, \end{array}$$ (13)

where $W_{3}\in {\mathbb{R}}^{256\times D_{\text{f}}}$, $W_{4}\in {\mathbb{R}}^{C\times 256}$, and **b**_3_, **b**_4_ are learnable parameters.

Compared with the baseline, MAM explicitly ties the dermoscopic representation to metadata through attention, enabling the classifier to adapt its decision boundary according to clinical context.

### 3.3 Class-aware optimization (CAO)

The ISIC 2019 dataset exhibits a pronounced class imbalance, with common benign lesions dominating the training set, while certain malignant categories and rare benign types contain far fewer examples. Direct training with standard mini-batch sampling and uniform cross-entropy easily leads to biased decision boundaries and poor sensitivity on under-represented classes. To alleviate this issue, we introduce a class-aware optimization component that combines balanced sampling and parameter stabilization via EMA.

#### 3.3.1 Class-balanced sampling

Let *N*_*c*_ denote the number of training samples belonging to class *c*, and let $N=\overset{C-1}{\underset{c=0}{\sum }}N_{c}$ be the total number of training samples.

We first define inverse-frequency class weights, as shown in Equation 14:


$$\begin{array}{l} w_{c}^{\text{base}}=\frac{N}{C\cdot N_{c}},\;c=0,\ldots ,C-1, \end{array}$$ (14)

which assign larger weights to classes with fewer samples. These base weights are normalized such that their average equals 1, as shown in Equation 15:


$$\begin{array}{l} \frac{1}{C}\overset{C-1}{\underset{c=0}{\sum }}w_{c}^{\text{base}}=1. \end{array}$$ (15)

During data loading, each sample is assigned a sampling weight equal to $w_{c}^{\text{base}}$ based on its ground-truth label. We then use WeightedRandomSampler with replacement to construct mini-batches. Intuitively, this procedure increases the frequency with which minority classes appear in training batches, while still preserving the overall label distribution.

#### 3.3.2 Exponential Moving Average (EMA)

Training a large ViT backbone on a highly imbalanced dataset may lead to unstable updates and overfitting. To improve robustness, we maintain an EMA of the model parameters. Let θ_*t*_ denote the current network parameters at optimization step *t*, and let $\theta_{t}^{\text{EMA}}$ be the corresponding EMA parameters. At each step, we update as shown in Equation 16:


$$\begin{array}{l} \theta_{t}^{\text{EMA}}=\text{λ}\theta_{t-1}^{\text{EMA}}+\left(1-\text{λ}\right)\theta_{t}, \end{array}$$ (16)

where λ ∈ (0, 1) is the decay factor, set to λ = 0.999 in our experiments. The EMA parameters are used for validation and test-time inference, while the original parameters θ_*t*_ continue to be updated by back-propagation. This strategy smooths noisy updates and often yields better generalization on minority classes.

### 3.4 UNK-aware decision rule (UDR)

The official ISIC 2019 test set contains an additional “unknown” (UNK) category that does not appear in the training set. Our model is trained only on the *C* = 8 known classes and does not explicitly output a logit for UNK. To handle this mismatch at evaluation time, we design a simple yet effective unknown-aware decision rule.

Given the predicted probabilities over known classes, as shown in Equation 17:


$$\begin{array}{l} p=\text{softmax}\left(\ell \right)\in {\mathbb{R}}^{C}, \end{array}$$ (17)

we compute the maximum confidence, as shown in Equation 18:


$$\begin{array}{l} p_{\max}=\underset{c\in \left\{0,\ldots ,C-1\right\}}{\max}p_{c}. \end{array}$$ (18)

If *p*_max_ is below a pre-defined threshold τ, we consider the sample unreliable for any known class and assign it to the UNK category.

Formally, the final prediction ŷ in the 9-class setting is given by Equation 19:


$$\hat{y}=\{\begin{array}{ll} C, & \text{if }p_{\max}<\tau ,\\ \arg\underset{c\in \{0,\ldots ,C-1\}}{\max}p_{c}, & \text{otherwise}, \end{array}$$ (19)

where index *C* corresponds to UNK, and τ ∈ (0, 1) is a hyperparameter (set to 0.6 by default). This UDR encourages the model to abstain on highly uncertain cases, improving robustness in the presence of unseen lesion types.

### 3.5 Loss function

We optimize the entire network using a label-smoothed, class-weighted cross-entropy loss defined over the *C* known classes. For a given input (**X**, **m**) with ground-truth label *y*, the model outputs logits ℓ ∈ ℝ^*C*^ and probabilities **p** = softmax(ℓ). We first construct a one-hot label vector ${\text{e}}_{y}\in {\left\{0,1\right\}}^{C}$ such that *e*_*y, c*_ = 1 if *c* = *y* and 0 otherwise. To alleviate overconfidence and improve calibration, we apply label smoothing with parameter ε, as shown in Equation 20:


$$\begin{array}{l} \tilde{y}=\left(1-\varepsilon \right)e_{y}+\frac{\varepsilon }{C}1, \end{array}$$ (20)

where **1** ∈ ℝ^*C*^ is an all-ones vector and ε = 0.05 in our implementation. Thus, the target distribution $\tilde{\text{y}}$ assigns a slightly lower probability to the ground-truth class and distributes the remaining mass uniformly over all classes.

In parallel, we derive class weights from the training set to mitigate label imbalance. Let *N*_*c*_ be the number of samples for class *c*, and *N* the total number of samples. We define base inverse-frequency weights as shown in Equation 21:


$$\begin{array}{l} w_{c}^{\text{base}}=\frac{N}{C\cdot N_{c}}. \end{array}$$ (21)

To further emphasize particularly challenging minority classes, we slightly boost the weights of the AK, BKL and SCC categories by a factor α_small_, as shown in Equation 22:


$$w_{c}=\{\begin{array}{ll} \alpha_{\text{small}}\cdot w_{c}^{\text{base}}, & c\in \{\text{AK},\text{BKL},\text{SCC}\},\\ w_{c}^{\text{base}}, & \text{otherwise}, \end{array}$$ (22)

with α_small_ = 1.2. Finally, the weights {*w*_*c*_} are normalized such that Equation 23:


$$\begin{array}{l} \frac{1}{C}\overset{C-1}{\underset{c=0}{\sum }}w_{c}=1. \end{array}$$ (23)

Given a mini-batch of size *B*, with logits ℓ^(*i*)^ and smoothed targets ${\tilde{\text{y}}}^{\left(i\right)}$ for sample *i*, the training objective is defined in Equation 24:


$$\begin{array}{l} L=-\frac{1}{B}\overset{B}{\underset{i=1}{\sum }}\overset{C-1}{\underset{c=0}{\sum }}w_{c}{ỹ}_{c}^{\left(i\right)}\log p_{c}^{\left(i\right)}, \end{array}$$ (24)

where $p_{c}^{\left(i\right)}$ is the predicted probability of class *c* for sample *i*. This loss jointly incorporates label smoothing and class re-weighting: label smoothing prevents the model from becoming overly confident and improves generalization, while class weights compensate for imbalance by penalizing errors on rare classes more strongly. Combined with the class-balanced sampling and EMA in CAO, the loss function forms a coherent class-aware optimization strategy tailored to the ISIC 2019 lesion distribution.

### 3.6 Class-balanced focal loss with Sqrt-CB sampling

While the inverse-frequency weighting and manual small-class boosting described in Section 3.5 provide a reasonable first-order correction for class imbalance, they have two limitations: (i) the hand-tuned boost factor α_small_ does not generalize well across datasets with different imbalance profiles, and (ii) standard cross-entropy treats all correctly classified samples equally, regardless of their prediction confidence, which wastes gradient budget on already well-learned examples. To address both issues, we replace the original loss with a class-balanced focal loss that combines the effective-number re-weighting of Cui et al. (11) with the focal modulation of Lin et al. (12).

#### 3.6.1 Class-balanced weighting

The effective number of samples for class *c* is defined as Equation 25:


$$\begin{array}{l} E_{c}=\frac{1-\beta^{N_{c}}}{1-\beta }, \end{array}$$ (25)

where β ∈ [0, 1) is a hyperparameter that controls the rate at which additional samples contribute diminishing marginal information. Setting β = 0 recovers uniform weighting, while β → 1 approaches inverse-frequency weighting. In practice, we set β = 0.999. The CB weight for class *c* is defined in Equation 26:


$$\begin{array}{l} w_{c}^{\text{CB}}=\frac{1}{E_{c}}=\frac{1-\beta }{1-\beta^{N_{c}}}. \end{array}$$ (26)

These weights are normalized so that $\underset{c}{\sum }w_{c}^{\text{CB}}=C$.

#### 3.6.2 Focal modulation

Following Lin et al. (12), we multiply the per-sample cross-entropy by a modulating factor ${\left(1-p_{t}\right)}^{\gamma }$, where *p*_*t*_ is the predicted probability of the ground-truth class and γ≥0 is the focusing parameter (set to γ = 2.0). This down-weights the loss contribution of easy, well-classified examples and focuses training on hard cases, as shown in Equation 27:


$$\begin{array}{l} L_{\text{CB-Focal}}=-\frac{1}{B}\overset{B}{\underset{i=1}{\sum }}w_{y^{\left(i\right)}}^{\text{CB}}{\left(1-p_{y^{\left(i\right)}}^{\left(i\right)}\right)}^{\gamma }\log p_{y^{\left(i\right)}}^{\left(i\right)}, \end{array}$$ (27)

where *y*^(*i*)^ is the ground-truth label of sample *i* and $p_{y^{\left(i\right)}}^{\left(i\right)}$ is the predicted probability for that class.

#### 3.6.3 Square-root CB sampling

To complement the loss-level re-weighting, we also modify the data sampling strategy. Instead of using raw inverse-frequency weights, we assign each sample a sampling probability proportional to $\sqrt{w_{c}^{\text{CB}}}$, which provides a softer upsampling of rare classes than fully balanced sampling. This prevents the model from seeing too many repeated copies of the rarest classes per epoch, reducing overfitting risk while still substantially increasing their representation compared to natural sampling.

### 3.7 Patch-level cross-attention MAM

The original MAM (Section 3.2) projects the CLS token—a single global image summary—into the fusion space and performs self-attention with the metadata token. While effective, this design discards the rich spatial information encoded in the $N_{p}={\left(384/16\right)}^{2}=576$ patch tokens output by the ViT backbone. Certain metadata fields, notably anatomical site, carry spatially relevant information (e.g., lesions on palms/soles exhibit distinctive dermatoglyphic patterns), suggesting that allowing metadata to attend to local image regions could improve fusion quality.

We therefore introduce a patch-level cross-attention variant of MAM. Let ${\text{Z}}_{\text{patch}}\in {\mathbb{R}}^{N_{p}\times D_{\text{img}}}$ denote the sequence of patch token embeddings from the ViT backbone (excluding the CLS token). We project these into the fusion space, as shown in Equation 28:


$$\begin{array}{l} T_{\text{patch}}=Z_{\text{patch}}W_{\text{patch}}^{⊤}+1_{N_{p}}b_{\text{patch}}^{⊤}\in {\mathbb{R}}^{N_{p}\times D_{\text{f}}}, \end{array}$$ (28)

where $W_{\text{patch}}\in {\mathbb{R}}^{D_{\text{f}}\times D_{\text{img}}}$ and ${\text{b}}_{\text{patch}}\in {\mathbb{R}}^{D_{\text{f}}}$ are learnable parameters. The metadata token ${\text{t}}_{\text{meta}}\in {\mathbb{R}}^{D_{\text{f}}}$ is computed as before.

We then perform multi-head cross-attention where the metadata token serves as the query and the patch tokens serve as keys and values, as shown in Equation 29:


$$\begin{array}{l} t_{\text{meta}}^{\prime }=\text{MHA}\left(t_{\text{meta}},T_{\text{patch}},T_{\text{patch}}\right)\in {\mathbb{R}}^{D_{\text{f}}}, \end{array}$$ (29)

using 4 attention heads. This allows the metadata embedding to selectively attend to the most relevant spatial regions of the dermoscopic image. The resulting metadata-enriched token ${\text{t}}_{\text{meta}}^{\prime }$ is then combined with the projected CLS token **t**_img_ via average pooling and layer normalization, analogous to the original MAM, as shown in Equation 30:


$$\begin{array}{l} z_{\text{fused}}=\text{LN}\left(\frac{t_{\text{img}}+t_{\text{meta}}^{\prime }}{2}\right)\in {\mathbb{R}}^{D_{\text{f}}}. \end{array}$$ (30)

The fused representation **z**_fused_ is then passed to the same classification head as before. By grounding metadata in spatially localized image features, patch-level cross-attention MAM can capture finer correlations between clinical context and visual patterns.

### 3.8 Supervised contrastive regularization

The classification loss optimizes the decision boundary in logit space, but does not directly shape the geometry of the learned feature representations. In the highly imbalanced skin lesion setting, representations of rare classes may occupy diffuse regions in the embedding space, making them vulnerable to decision-boundary shifts caused by dominant classes. To address this, we add a supervised contrastive (SupCon) regularization term (13) that operates on the fused representations **z**_fused_.

For each sample *i* in a mini-batch, we first ℓ_2_-normalize the fused representation to obtain ${\text{v}}^{\left(i\right)}={\text{z}}_{\text{fused}}^{\left(i\right)}/||{\text{z}}_{\text{fused}}^{\left(i\right)}{||}_{2}$. Let $P\left(i\right)=\left\{j\neq i:y^{\left(j\right)}=y^{\left(i\right)}\right\}$ denote the set of indices of other samples in the batch sharing the same class as sample *i*, and let |P(i)| be its cardinality. The SupCon loss for the batch is defined as Equation 31:


$$\begin{array}{l} L_{\text{SupCon}}=-\frac{1}{B}\overset{B}{\underset{i=1}{\sum }}\frac{1}{\left|P\left(i\right)\right|}\underset{j\in P\left(i\right)}{\sum }\log\frac{\exp\left(v^{\left(i\right)}\cdot v^{\left(j\right)}/\tau_{c}\right)}{\sum_{k\neq i}\exp\left(v^{\left(i\right)}\cdot v^{\left(k\right)}/\tau_{c}\right)}, \end{array}$$ (31)

where τ_*c*_ = 0.1 is a temperature hyperparameter. If |P(i)|=0 (i.e., the sample is the only representative of its class in the batch), the corresponding term is set to zero.

The overall training objective for the extended ISIC-ViT is defined in Equation 32:


$$\begin{array}{l} L_{\text{total}}=L_{\text{CB-Focal}}+{\text{λ}}_{\text{sc}}L_{\text{SupCon}}, \end{array}$$ (32)

where λ_sc_ = 0.1 controls the relative weight of the contrastive regularizer. By encouraging representations of the same class to cluster together and representations of different classes to be pushed apart, the SupCon term improves the geometric quality of the embedding space, which in turn helps the linear classification head draw cleaner decision boundaries, especially between visually similar lesion categories.

## 4 Experiments

### 4.1 Datasets and evaluation metrics

#### 4.1.1 ISIC 2019 skin lesion classification challenge

We evaluate our method on the official ISIC 2019 skin lesion classification benchmark, which consists of dermoscopic images collected from multiple publicly available sources, including HAM10000, BCN 20000, and MSK. All images are RGB dermoscopic photographs with varying resolutions, and the dataset is released under a CC BY-NC 4.0 license for non-commercial research use. Each image is assigned to one of nine diagnostic categories: melanoma (MEL), nevus (NV), basal cell carcinoma (BCC), actinic keratosis (AK), benign keratosis (BKL), dermatofibroma (DF), vascular lesions (VASC), squamous cell carcinoma (SCC), and an additional unknown category (UNK) that only appears in the test set. The official training split contains 25,331 images, while the test split contains 8,238 images with hidden labels. Following the challenge protocol, we use the official ground-truth CSV files to retrieve labels for all experiments.

The training set is highly imbalanced. NV accounts for more than half of the training images, whereas rare classes such as DF and VASC contain fewer than 300 samples. In contrast, the test set includes a large number of UNK images (2,047 samples), which need to be handled explicitly at evaluation time. In addition to image labels, ISIC 2019 provides structured metadata for each sample, including approximate age, patient sex, and anatomical site. These metadata fields are used as auxiliary inputs in our model.

#### 4.1.2 BCN 20000 dataset

To evaluate the generalizability of our proposed improvements, we additionally conduct experiments on the BCN 20000 dataset, which is one of the primary data sources for ISIC 2019. The BCN 20000 training split contains 12,413 dermoscopic images labeled with the same 8 diagnostic categories as the known ISIC 2019 classes (MEL, NV, BCC, AK, BKL, DF, VASC, SCC), but does not include a UNK category. The class distribution follows a similar long-tailed pattern, with NV (33.9%) and MEL (23.0%) dominating, while DF (1.0%) and VASC (0.9%) are extremely rare. Metadata fields include sex, anatomical site, approximate age, lesion ID, and capture date. We use BCN 20000 as a secondary benchmark to verify that the proposed extensions generalize beyond the ISIC 2019 data mixture.

#### 4.1.3 Train/validation splits

For model development on ISIC 2019, we adopt the official training split for supervised learning and hold out a validation set from the training data to tune hyperparameters. Specifically, we group images by the lesion identifier so that all images from the same lesion share a unique lesion ID. We then perform a group-wise split at the lesion level to prevent images of the same lesion from appearing in both training and validation sets, which would otherwise lead to optimistic estimates. Following this protocol, 85% of the lesions are used for training and the remaining 15% are used for validation, resulting in approximately 21,437 training images and 3,894 validation images. For BCN 20000, we similarly apply a lesion-ID-based group-wise split with the same 85/15 ratio, yielding approximately 10,578 training images and 1,835 validation images. The official ISIC 2019 test split with 8,238 images is kept untouched during training and is used only for the final baseline comparison (Table 1).

**Table 1:** Comparison with CNN and transformer baselines on the ISIC 2019 test set.

Method	Backbone	Meta	ACC	Macro AUC	Macro BACC	WBACC _score_	WBACC _val_
VGG16-BN (19)	VGG16-BN	✗	0.5443	0.8783	0.7388	0.7154	0.8214
ResNet-18 (20)	ResNet-18	✗	0.5094	0.8618	0.3599	0.7266	0.8168
ResNet-50 (20)	ResNet-50	✗	0.5149	0.8620	0.7435	0.7399	0.8258
DenseNet-121 (21)	DenseNet-121	✗	0.1496	0.7671	0.6231	0.6220	0.6060
EfficientNet-B0 (22)	Eff-B0	✗	0.0686	0.6606	0.5633	0.5652	0.4790
ConvNeXt-Tiny (23)	ConvNeXt-T	✗	0.0573	0.6949	0.5748	0.5746	0.5714
Swin-Tiny (24)	Swin-T	✗	0.2017	0.7763	0.6392	0.6373	0.6497
ViT (17)	ViT-B/16	✗	0.5601	0.7830	0.6480	0.6460	0.6730
**Ours**	ViT-B/16	✓	**0.7023**	**0.8849**	**0.7543**	**0.7510**	**0.8481**

We report overall accuracy (ACC), macro-averaged AUC (MacroAUC), macro-averaged balanced accuracy (MacroBACC), and weighted balanced accuracy computed with the official score_weight and validation_weight. Bold values indicate the best performance in each column.

Since the UNK category does not appear in the training labels, our network is trained as an 8-class classifier over the known categories (i.e., MEL, NV, BCC, AK, BKL, DF, VASC, SCC). For the ablation study and extended model evaluation, we report results on the held-out validation sets of both ISIC 2019 (8-class, no UNK) and BCN 20000 (8-class).

#### 4.1.4 Evaluation metrics

We follow the ISIC challenge protocol and recent works on skin lesion classification, and adopt several complementary metrics to comprehensively assess performance. Let ${\left\{\left(x_{i},y_{i},w_{i}\right)\right\}}_{i=1}^{N}$ denote the evaluation set, where *y*_*i*_ ∈ {0, …, *C*} is the ground-truth label (with *C* = 7 in the 8-class setting and *C* = 8 in the 9-class setting), and *w*_*i*_ is an optional non-negative sample weight derived from the official score_weight or validation_weight fields. For each sample, the model outputs a predicted label ŷ_*i*_ and class probabilities.

The overall (unweighted) accuracy is defined as Equation 33:


$$\begin{array}{l} \text{ACC}=\frac{1}{N}\overset{N}{\underset{i=1}{\sum }}1\left({ŷ}_{i}=y_{i}\right), \end{array}$$ (33)

where 1(·) is the indicator function that equals 1 if the argument is true and 0 otherwise.

To better capture performance under severe class imbalance, we compute class-wise sensitivity and specificity. For each class *c*, let TP_*c*_, TN_*c*_, FP_*c*_ and FN_*c*_ denote the numbers of true positives, true negatives, false positives and false negatives under a one-vs.-rest formulation. The sensitivity (recall) and specificity of class *c* are given by Equation 34:


$$\begin{array}{l} \text{S}{\text{E}}_{c}=\frac{\text{T}{\text{P}}_{c}}{\text{T}{\text{P}}_{c}+\text{F}{\text{N}}_{c}},\text{ S}{\text{P}}_{c}=\frac{\text{T}{\text{N}}_{c}}{\text{T}{\text{N}}_{c}+\text{F}{\text{P}}_{c}}. \end{array}$$ (34)

The balanced accuracy of class *c* is defined in Equation 35:


$$\begin{array}{l} \text{BAC}{\text{C}}_{c}=\frac{\text{S}{\text{E}}_{c}+\text{S}{\text{P}}_{c}}{2}, \end{array}$$ (35)

and the macro balanced accuracy over all *C*+1 classes (including UNK in the 9-class setting) is computed as shown in Equation 36:


$$\begin{array}{l} \text{MacroBACC}=\frac{1}{C+1}\overset{C}{\underset{c=0}{\sum }}\text{BAC}{\text{C}}_{c}. \end{array}$$ (36)

In addition, we report the one-vs.-rest area under the ROC curve (AUC) for each class. Given the predicted probabilities for class *c* and the corresponding binary labels, we compute AUC_*c*_ and then take the arithmetic mean over all classes, as shown in Equation 37:


$$\begin{array}{l} \text{MacroAUC}=\frac{1}{C+1}\overset{C}{\underset{c=0}{\sum }}\text{AU}{\text{C}}_{c}. \end{array}$$ (37)

Therefore, our main comparison metrics include overall accuracy (ACC), macro AUC, and macro balanced accuracy (MacroBACC). These metrics together provide a comprehensive and fair evaluation under the highly imbalanced and clinically critical ISIC 2019 setting.

### 4.2 Implementation details

In all experiments, we implement our framework in PyTorch using the ViT-Base/16 backbone (vit_base_patch16_384) from the timm library, pre-trained on ImageNet. During pre-processing, each dermoscopic image is converted to RGB and resized to a network input of 384 × 384 pixels. We do *not* use zero padding: the short side of the original image is bilinearly resized to 1.1 × 384 = 422 pixels while preserving the aspect ratio, after which a 384 × 384 center crop is taken (for validation and test) or a RandomResizedCrop with scale range (0.8, 1.0) is applied (for training). Pixel values are then normalized using the standard ImageNet mean/standard-deviation statistics (μ = [0.485, 0.456, 0.406], σ = [0.229, 0.224, 0.225]). This protocol therefore trims a small fraction of pixels from the longer image dimension rather than padding zeros around the lesion; the structured metadata (sex, anatomical site, and approximate age) are encoded and fused with image features as described in Section 3. We optimize the network with AdamW, setting the learning rate of the ViT backbone to 5.0 × 10^−5^ and that of the metadata encoder, fusion module, and classifier head to 5.0 × 10^−4^, with a weight decay of 5.0 × 10^−2^. The learning rate is linearly warmed up during the first 5 epochs and then decayed to zero with a cosine schedule over a total of 60 epochs. We use a batch size of 32 and the training-time augmentations described above (random resized crop, horizontal/vertical flip, color jitter and random erasing with *p* = 0.25). An EMA of the model parameters is maintained during training with decay λ = 0.999, and all reported results are obtained from the EMA model.

For the baseline and original ISIC-ViT (Table 1), we use WeightedRandomSampler together with a class-weighted cross-entropy loss with label smoothing (ε = 0.05). For the extended model (Tables 2, 3), we replace the original loss with CB focal loss (β = 0.999, γ = 2.0), use square-root CB sampling, enable patch-level cross-attention MAM, and add SupCon regularization (λ_sc_ = 0.1, τ_*c*_ = 0.1). The contrastive temperature τ_*c*_ is fixed throughout training. Early stopping with patience of 15 epochs (based on validation MacroAUC) is applied to all extended experiments.

**Table 2:** Ablation study of the three proposed extensions on the BCN 20000 and ISIC 2019 validation sets.

	BCN 20000 val (*n* = 1, 835)			ISIC 2019 val (*n* = 3, 891)		
Configuration	ACC	MacroAUC	MacroBACC	ACC	MacroAUC	MacroBACC
Baseline	0.6327	0.8720	**0.7662**	0.4854	0.9086	0.7957
+CBFL	**0.6943**	**0.9228**	0.7500	0.7892	0.9587	0.8239
+CBFL+PMAM	0.6812	0.9179	0.7506	0.7756	0.9573	0.8415
+CBFL+PMAM+SupCon	0.6943	0.9074	0.7587	**0.7961**	**0.9613**	**0.8426**

Baseline: original ISIC-ViT (MAM + CAO + EMA). +CBFL: Class-Balanced Focal Loss with sqrt-CB sampling. +PMAM: Patch-level cross-attention MAM. +SupCon: Supervised contrastive regularization. All results are from EMA models. Bold values indicate the best performance in each column.

**Table 3:** Per-class diagnostic performance on both datasets: Baseline (original ISIC-ViT) vs. Improved (+CBFL+PMAM+SupCon).

	BCN 20000 val						ISIC 2019 val					
	Baseline			Improved			Baseline			Improved		
Class	SE	AUC	BACC	SE	AUC	BACC	SE	AUC	BACC	SE	AUC	BACC
MEL	0.568	0.868	0.745	0.665^↑^	0.906^↑^	0.796^↑^	0.641	0.771	0.688	0.562^↓^	0.899^↑^	0.764^↑^
NV	0.710	0.914	0.819	0.821^↑^	0.918^↑^	0.843^↑^	0.350	0.924	0.671	0.929^↑^	0.964^↑^	0.894^↑^
BCC	0.678	0.913	0.802	0.756^↑^	0.949^↑^	0.843^↑^	0.626	0.949	0.791	0.858^↑^	0.983^↑^	0.910^↑^
AK	0.562	0.840	0.753	0.500^↓^	0.915^↑^	0.731^↓^	0.556	0.905	0.751	0.416^↓^	0.954^↑^	0.699^↓^
BKL	0.488	0.806	0.710	0.362^↓^	0.839^↑^	0.666^↓^	0.564	0.866	0.752	0.682^↑^	0.944^↑^	0.824^↑^
DF	0.393	0.763	0.682	0.393	0.853^↑^	0.693^↑^	0.920	0.979	0.918	0.880^↓^	0.991^↑^	0.935^↑^
VASC	0.850	0.993	0.916	0.600^↓^	0.967^↓^	0.797^↓^	1.00	0.999	0.989	0.875^↓^	0.999	0.937^↓^
SCC	0.447	0.879	0.702	0.434^↓^	0.914^↑^	0.701^↓^	0.659	0.876	0.806	0.571^↓^	0.956^↑^	0.779^↓^
*Avg*	*0.587*	*0.872*	*0.766*	*0.566^↓^*	*0.908^↑^*	*0.759^↓^*	*0.665*	*0.909*	*0.796*	*0.722^↑^*	*0.961^↑^*	*0.843^↑^*

All results are from EMA models. SE: sensitivity, AUC: one-vs-rest area under the ROC curve, BACC: balanced accuracy. In the Improved columns, ^↑^ (^↓^) marks values that are strictly above (below) the corresponding Baseline within the same dataset; ties are unmarked. See Section 4.5 for details. Italic values represent average results.

#### 4.2.1 Software and hardware environment

For reproducibility, we provide the exact software stack and hardware platform used to produce the reported results. The code is written in Python 3.13.11 (Python Software Foundation, Beaverton, OR, USA) and depends on PyTorch 2.9.1 [PyTorch Foundation (Linux Foundation), San Francisco, CA, USA] compiled against CUDA 12.8 with cuDNN 9.10.2, together with torchvision 0.24.1, timm 1.0.25 and NumPy 2.3.5. All experiments use a single global random seed of 42, applied identically to Python's built-in random module, NumPy's np.random generator (legacy Mersenne-Twister state), and PyTorch's CPU and CUDA RNGs via torch.manual_seed and torch.cuda.manual_seed_all; for deterministic kernel selection we additionally set torch.backends.cudnn.deterministic = True and torch.backends.cudnn.benchmark = False. The same seed controls the lesion-ID group-wise train/validation split (via sklearn.GroupShuffleSplit) so that all configurations in our ablations share an identical 85/15 split. Training is performed on a workstation with an NVIDIA GeForce RTX 4090 GPU (24 GB GDDR6X, NVIDIA driver 550 series), an AMD Ryzen multi-core CPU and 64 GB of DDR5 system memory, running Ubuntu 22.04 LTS with Linux kernel 5.15. Inference for evaluation is performed in mixed-precision with the same software stack. With the seed, software versions, hardware platform, and pre-processing protocol specified above, the entire pipeline—from raw ISIC/BCN data through to the reported metrics—is fully described, and any remaining implementation details can be obtained from the corresponding author upon reasonable request.

### 4.3 Comparison to other methods

We compare the proposed ViT-based multi-modal classifier with a set of representative CNN and transformer baselines on the ISIC 2019 test set, as summarized in Table 1. All models are trained under the same data split and augmentation protocol described in Section 4.2, and, unless otherwise specified, take only the dermoscopic image as input. Classical CNN backbones such as VGG16-BN and ResNet-50 obtain ACC around 0.51–0.54 and MacroBACC around 0.74, while more recent architectures including DenseNet-121, EfficientNet-B0, ConvNeXt-Tiny, and Swin-Tiny perform noticeably worse in this highly imbalanced setting, with ACC dropping below 0.21 and MacroBACC below 0.64. A vanilla ViT-B/16 model using image-only input reaches 0.5601 ACC and 0.6480 MacroBACC, which is comparable to strong CNN baselines but still lags behind in terms of balanced accuracy. In contrast, our full model (ViT-B/16 + Meta) fuses dermoscopic images with patient-level metadata via cross-modal attention and clearly outperforms all competitors. It achieves an ACC of 0.7023, a MacroAUC of 0.8849, and a MacroBACC of 0.7543, together with the best weighted balanced accuracies (WBACC_*score*_ = 0.7510 and WBACC_*val*_ = 0.8481). These results indicate that metadata provides information that is complementary to the image stream, and that our fusion and training strategy is more effective than simply changing the backbone architecture on this clinically imbalanced classification task.

### 4.4 Ablation study

To systematically evaluate the contribution of each proposed extension, we conduct an ablation study on both the BCN 20000 validation set (1,835 samples, 8 classes) and the ISIC 2019 validation set (3,894 samples, 8 classes), as shown in Table 2. The baseline corresponds to the original ISIC-ViT with MAM, CAO, and EMA. We then incrementally add the three extensions: CBFL (Class-Balanced Focal Loss with sqrt-CB sampling), PMAM (Patch-level cross-attention MAM), and SupCon (Supervised contrastive regularization).

On the BCN 20000 validation set, adding CB focal loss (+CBFL) yields a large improvement in ACC (from 0.6327 to 0.6943, a 6.2 percentage point gain) and MacroAUC (from 0.8720 to 0.9228, a 5.1 point gain), demonstrating that the CB focal loss provides substantially better handling of the long-tailed distribution compared to the original inverse-frequency weighting. Adding patch-level cross-attention (+CBFL+PMAM) slightly reduces ACC to 0.6812 and MacroAUC to 0.9179 while keeping MacroBACC essentially unchanged at 0.7506. The full model (+CBFL+PMAM+SupCon) recovers ACC to 0.6943 (matching +CBFL) and lifts MacroBACC back to 0.7587. Interestingly, the highest MacroBACC on BCN 20000 is in fact attained by the *Baseline* configuration (0.7662), which slightly exceeds the best extended variant (0.7587 for +CBFL+PMAM+SupCon). We attribute this to the smaller training pool of BCN 20000 (~10.6k images) relative to ISIC 2019 (~21.4k): the aggressive inverse-frequency oversampling used by the baseline boosts per-class sensitivity for rare categories at the cost of overall ranking quality (MacroAUC 0.8720 vs. 0.9074–0.9228 for the extended models), whereas the proposed extensions trade a small amount of MacroBACC for substantially better MacroAUC and ACC. The implication of this dataset-size-dependent behavior is further discussed in Section 4.6.

On the ISIC 2019 validation set, all three extensions contribute positively, and the full model consistently achieves the best results. The baseline starts at 0.4854 ACC, 0.9086 MacroAUC, and 0.7957 MacroBACC. Adding CB focal loss (+CBFL) brings a dramatic ACC improvement to 0.7892 (+30.4 points), MacroAUC to 0.9587 (+5.0 points), and MacroBACC to 0.8239 (+2.8 points). Adding patch-level cross-attention (+CBFL+PMAM) further lifts MacroBACC to 0.8415, indicating that the finer-grained spatial fusion is beneficial for class-balanced performance. The full model (+CBFL+PMAM+SupCon) achieves the best scores across all three metrics: 0.7961 ACC, 0.9613 MacroAUC, and 0.8426 MacroBACC. Compared to the baseline, this represents improvements of +31.1 points in ACC, +5.3 points in MacroAUC, and +4.7 points in MacroBACC.

### 4.5 Per-class analysis

Table 3 presents a detailed per-class comparison between the baseline and improved (+CBFL+PMAM+SupCon) models on both datasets, while Table 4 quantifies the incremental contribution of each extension. To make Table 3 easy to read at a glance and avoid any ambiguity from cell-level bold formatting, we annotate each *Improved*-column value with a directional symbol: ↑ marks metrics on which the Improved model *strictly* outperforms the Baseline, ↓ marks metrics on which it *strictly* underperforms, and equal values carry no symbol. The Baseline column is always shown without any annotation. Counting ↑ entries in the body of the table, the Improved model wins on 14/24 metric–class combinations on BCN 20000 and 15/24 on ISIC 2019, with the wins concentrated in AUC (7/8 classes on each dataset, the single exception being the rare VASC class) and in the four largest classes (MEL, NV, BCC, BKL on ISIC 2019; NV, BCC on BCN 20000). The ↓ entries correspond to the SE–BACC trade-offs analyzed in the remainder of this section and in Section 4.6.

**Table 4:** Incremental contribution of each proposed extension (EMA evaluation).

	BCN 20000		ISIC 2019	
Step	ΔACC	ΔMacroAUC	ΔACC	ΔMacroAUC
Baseline → +CBFL	+6.2	+5.1	+30.4	+5.0
+CBFL → +CBFL+PMAM	−1.3	−0.5	−1.4	−0.1
+CBFL+PMAM → +CBFL+PMAM+SupCon	+1.3	−1.0	+2.1	+0.4
*Total*	+*6.2*	+*3.5*	+*31.1*	+*5.3*

Δ values show the change from the previous configuration. Italic values indicate the total change from the Baseline to the final model.

#### 4.5.1 Per-class AUC improvement

**Figure 3:**
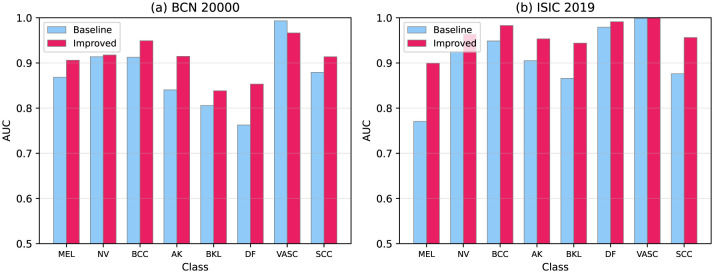
Per-class AUC comparison between baseline and improved (+CBFL+PMAM+SupCon) models on **(a)** BCN 20000 and **(b)** ISIC 2019 validation sets (EMA evaluation).

The improved model achieves a higher AUC than the baseline in nearly all classes on both datasets. On ISIC 2019, the most notable gains are MEL (0.771 → 0.899, +12.8 pp), BKL (0.866 → 0.944, +7.8 pp), and SCC (0.876 → 0.956, +8.0 pp). On BCN 20000, DF (0.763 → 0.853, +9.0 pp) and AK (0.840 → 0.915, +7.5 pp) show the largest gains. These consistent improvements across both datasets confirm that the proposed extensions enhance discriminative ability for both common and rare classes. Figure 3 visualizes the per-class AUC comparison.

#### 4.5.2 Sensitivity trade-offs

While AUC improves broadly, sensitivity (SE) shows a more nuanced pattern. On ISIC 2019, the improved model substantially increases SE for NV (0.350 → 0.929), BCC (0.626 → 0.858), and BKL (0.564 → 0.682), but decreases SE for AK (0.556 → 0.416) and MEL (0.641 → 0.562). A similar trade-off is observed on BCN 20000: the improved model wins on NV, BCC, and MEL (e.g., NV SE 0.710 → 0.821, MEL SE 0.568 → 0.665), while the Baseline retains higher SE on AK (0.562 vs. 0.500), BKL (0.488 vs. 0.362), VASC (0.850 vs. 0.600) and SCC (0.447 vs. 0.434). Averaged over all classes, the Improved model on BCN 20000 has slightly lower mean SE/BACC (0.566/0.759) than the Baseline (0.587/0.766) but markedly higher mean AUC (0.908 vs. 0.872). This reflects the removal of aggressive inverse-frequency oversampling: the baseline's extreme oversampling of rare classes artificially inflated their SE at the cost of overall discriminability, while the improved model achieves better calibrated ranking (higher AUC) with a more natural class distribution. The effect is more pronounced on the smaller BCN 20000 set, where extended modules have less data to converge against, whereas on the larger ISIC 2019 set the Improved model wins on both AUC *and* mean BACC.

#### 4.5.3 Confusion patterns

**Figure 4:**
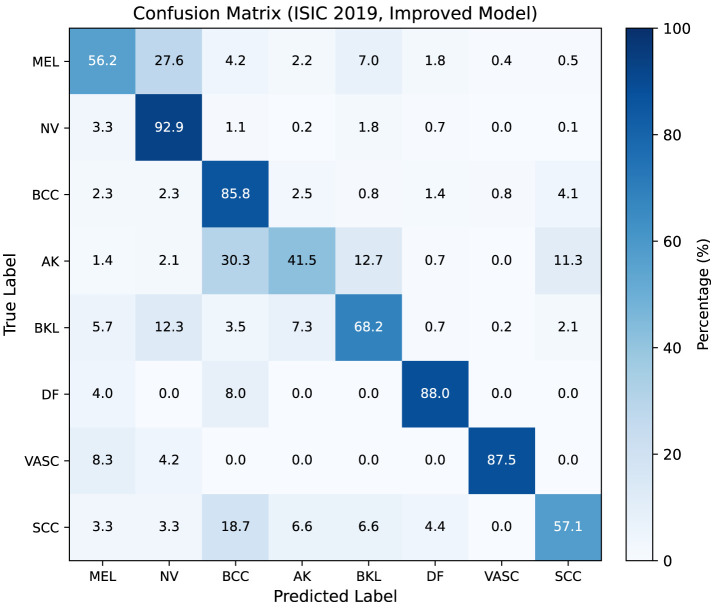
Normalized confusion matrix of the improved ISIC-ViT (+CBFL+PMAM+SupCon) on the ISIC 2019 validation set (EMA model). Each row is normalized to percentages of the true class. AK is most often confused with BCC (30.3%).

Figure 4 shows the confusion matrix for the improved model on ISIC 2019. The strongest diagonal entries are NV (92.9%), DF (88.0%), VASC (87.5%), and BCC (85.8%). The most common confusion pattern is AK being misclassified as BCC (30.3% of AK samples), which is clinically expected given that both are keratotic lesions with overlapping morphological features. MEL is occasionally confused with NV (14.2%) and BKL (11.9%), reflecting the well-known diagnostic difficulty of distinguishing melanoma from benign melanocytic and seborrheic lesions.

#### 4.5.4 Incremental contributions

Table 4 shows that CBFL (CB focal loss + sqrt-CB sampling) provides the largest single improvement on both datasets: +5.1 pp MacroAUC on BCN 20000 and +5.0 pp on ISIC 2019. PMAM (patch-level cross-attention) has a modest effect on aggregate metrics (−0.5 and −0.1 pp MacroAUC), but contributes meaningfully to per-class improvements for rare classes such as DF (Table 3). SupCon improves ACC on both datasets (+1.3 and +2.1 pp) and improves MacroAUC on ISIC 2019 (+0.4 pp), consistent with its role as a feature-space regularizer that helps balance the final classification decisions.

### 4.6 Discussion

#### 4.6.1 Dataset size and module effectiveness

An interesting pattern emerges from the ablation results across the two datasets. On BCN 20000 (12,413 training samples), adding CB focal loss alone (+CBFL) achieves the best MacroAUC (0.9228), while the original *Baseline* retains the best MacroBACC (0.7662); the subsequent addition of patch-level cross-attention and SupCon regularization (+CBFL+PMAM+SupCon) slightly decreases MacroAUC to 0.9074 but partially recovers MacroBACC from 0.7500 (+CBFL) to 0.7587, without surpassing the Baseline on this particular metric. In contrast, on ISIC 2019 (25,331 training samples), the full model consistently achieves the best performance across all three metrics, including MacroBACC (0.8426 vs. 0.7957 for the Baseline). A plausible explanation is that the additional modules—particularly patch-level cross-attention with its 576 key-value pairs and the contrastive loss with its $O\left(B^{2}\right)$ pairwise comparisons—require a sufficient volume of training data to learn meaningful representations without overfitting. The smaller BCN 20000 set may not provide enough diversity for these components to fully outweigh the simpler but heavily oversampled Baseline on per-class balanced accuracy, whereas the larger ISIC 2019 training set does.

#### 4.6.2 Baseline ACC discrepancy

The baseline ACC on the ISIC 2019 validation set (0.4854) is noticeably lower than the test-set ACC of the original study (0.7023). This discrepancy arises from several factors. First, the validation set contains only the 8 known classes and does not include UNK samples, resulting in a different class composition than the test set. Second, different random seeds and data splits lead to different training/validation partitions. Third, the original study's own ablation study showed that the full model achieved ACC = 0.6808 on the test set (Table 2 of the original study, “Full (M+A+C+E)” configuration), which is already lower than the best reported ACC. The validation-set evaluation provides a controlled, reproducible setting for comparing model variants, even though its absolute numbers differ from the test set.

#### 4.6.3 Validation vs. test set comparability

The validation set (8-class, no UNK) and the test set (9-class, with 2,047 UNK samples) measure different aspects of model performance and are not directly comparable. The test set additionally evaluates the UDR, which can shift overall ACC substantially depending on the confidence threshold τ. Our improvements are validated by consistent gains over our own baseline under identical evaluation conditions (same split, same metrics, same EMA evaluation), which provides a fair assessment of the relative contribution of each proposed component.

#### 4.6.4 Class weighting strategies

**Figure 5:**
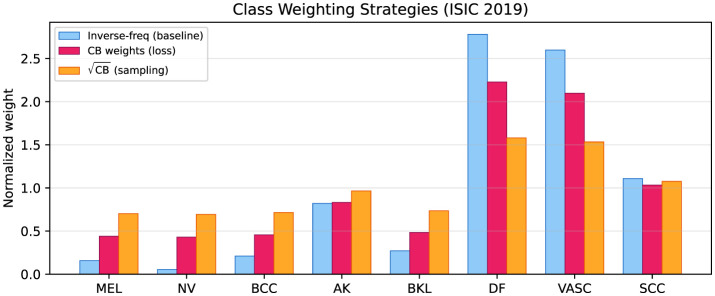
Comparison of class weighting strategies on ISIC 2019. The inverse-frequency weights (baseline) exhibit a 51.0 × max/min ratio, while CB weights and $\sqrt{\text{CB}}$ sampling weights reduce it to 5.2 × and 2.3 × , respectively.

Figure 5 compares the three weighting strategies used in our framework. The baseline's inverse-frequency weights exhibit extreme variation (51.0 × between the heaviest and lightest classes on ISIC 2019), which causes gradient domination by a handful of rare-class samples. The CB weights reduce this ratio to 5.2 × , providing smoother loss-level rebalancing. The square-root CB sampling weights further compress the ratio to 2.3 × , offering gentle upsampling of rare classes without the overfitting risk of full balanced sampling. The combination of CB loss weights with sqrt-CB sampling weights provides a principled two-tier rebalancing that is both theoretically grounded and empirically effective.

#### 4.6.5 AUC improvement vs. sensitivity shift

Across most classes, the extended model achieves substantially higher AUC compared to the baseline: for example, on ISIC 2019, MEL improves from AUC = 0.771 to 0.899 and SCC from 0.876 to 0.956. However, for some classes such as AK (SE = 0.416) and MEL (SE = 0.562), the sensitivity remains moderate despite high AUC values. This phenomenon reflects the distinction between ranking ability (measured by AUC) and hard classification (measured by argmax-based SE). The CB focal loss improves the model's probabilistic calibration and relative ordering of predictions, but the argmax decision boundary can still be shifted by the overwhelming majority class (NV, 50.8% of training data), causing some true rare-class samples to be narrowly outscored. Addressing this gap through class-specific threshold tuning or multi-stage classification remains an important direction for future work.

#### 4.6.6 Training efficiency

**Figure 6:**
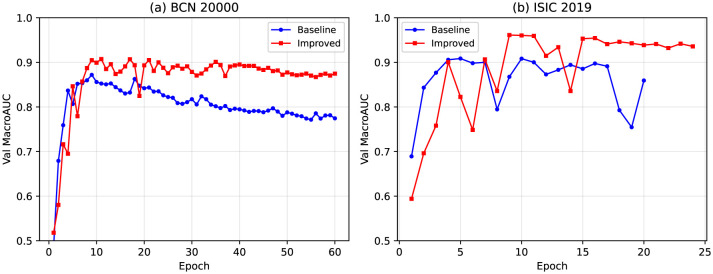
Validation MacroAUC curves for baseline (blue) and improved model (red) on **(a)** BCN 20000 and **(b)** ISIC 2019. The improved model achieves a higher peak MacroAUC on both datasets. Dashed vertical lines indicate the best epoch for each model.

Figure 6 shows the validation MacroAUC trajectories on both datasets. With early stopping (patience=15), the ISIC 2019 experiments converge in 20–25 epochs (best epoch 5–10), saving approximately 60% of the originally planned 60 epochs. On BCN 20000, experiments ran for the full 60 epochs without early stopping, but best performance was also reached in the first 8–18 epochs. The improved model reaches higher MacroAUC within fewer epochs on ISIC 2019 (best at epoch 9 vs. baseline best at epoch 5), suggesting that the CB focal loss and SupCon regularization improve both the convergence speed and the quality of the learned representations.

## 5 Conclusion

In this study, we proposed ISIC-ViT, a Vision Transformer-based framework tailored for the ISIC 2019 skin lesion classification task. It integrates dermoscopic images with simple but informative clinical metadata. Starting from a strong ViT-B/16 baseline, our method incorporates three key components: a metadata-guided cross-modal attention mechanism that fuses image and patient-level information into a unified representation, a class-aware optimization strategy combining balanced sampling with an EMA-stabilized backbone, and an UDR designed to handle the unseen “unknown” category at test time. Together with a label-smoothed, class-weighted cross-entropy loss, these components form a coherent training paradigm that targets the class imbalance and distributional heterogeneity inherent in the ISIC 2019 benchmark. On the official test set, ISIC-ViT consistently outperforms a diverse set of CNN and transformer baselines, reaching an ACC of 0.7023, a Macro AUC of 0.8849, and a Macro BACC of 0.7543.

Building on these foundations, we introduced three complementary extensions: class-balanced focal loss with square-root effective-number sampling, patch-level cross-attention MAM, and supervised contrastive regularization. Ablation experiments on the ISIC 2019 and BCN 20000 validation sets demonstrate that these extensions yield substantial and consistent improvements. On the ISIC 2019 validation set, the full extended model reaches an ACC of 0.7961, a Macro AUC of 0.9613, and a Macro BACC of 0.8426, representing gains of +31.1, +5.3, and +4.7 percentage points over the baseline, respectively. Per-class analysis confirms strong performance across both common and rare categories, with particularly notable gains for rare classes such as DF (AUC = 0.991, BACC = 0.935) and VASC (AUC = 0.999, BACC = 0.937).

The comparison between the two evaluation datasets further reveals that the full set of extensions is most effective when sufficient training data is available, as the smaller BCN 20000 dataset shows diminishing returns from the additional modules. In subsequent research, we plan to explore richer clinical metadata (e.g., longitudinal history, dermoscopic attribute scores), class-specific threshold optimization for improving sensitivity on rare malignant classes, and multi-task extensions that jointly model segmentation, calibration, and out-of-distribution detection. We believe that the design principles behind ISIC-ViT—namely, lightweight cross-modal fusion, modern loss design for long-tailed distributions, and simple but effective decision rules for unknown categories—are broadly applicable and can serve as a foundation for building more reliable, clinically oriented decision-support systems in dermatology and beyond.

## Data Availability

Data availability statement: Publicly available datasets were analyzed in this study. This data can be found here: The datasets analyzed in this study are publicly available. The ISIC 2019 dataset can be accessed through the ISIC Challenge Archive (https://challenge.isic-archive.com/data/), and the BCN20000 dataset is publicly available through the ISIC Archive and its associated public resources.
